# Condensin II Promotes the Formation of Chromosome Territories by Inducing Axial Compaction of Polyploid Interphase Chromosomes

**DOI:** 10.1371/journal.pgen.1002873

**Published:** 2012-08-30

**Authors:** Christopher R. Bauer, Tom A. Hartl, Giovanni Bosco

**Affiliations:** 1 Department of Molecular and Cellular Biology, The University of Arizona, Tucson, Arizona, United States of America; 2 Department of Genetics, The Geisel School of Medicine at Dartmouth, Dartmouth College, Hanover, New Hampshire, United States of America; Stowers Institute for Medical Research, United States of America

## Abstract

The eukaryotic nucleus is both spatially and functionally partitioned. This organization contributes to the maintenance, expression, and transmission of genetic information. Though our ability to probe the physical structure of the genome within the nucleus has improved substantially in recent years, relatively little is known about the factors that regulate its organization or the mechanisms through which specific organizational states are achieved. Here, we show that *Drosophila melanogaster* Condensin II induces axial compaction of interphase chromosomes, globally disrupts interchromosomal interactions, and promotes the dispersal of peri-centric heterochromatin. These Condensin II activities compartmentalize the nucleus into discrete chromosome territories and indicate commonalities in the mechanisms that regulate the spatial structure of the genome during mitosis and interphase.

## Author Summary

A number of recent studies have debunked the idea that chromosomes exist as a tangled mass of chromatin fibers within the nucleus. In many organisms, including mammals, each chromosome occupies a specific region of the nucleus known as a chromosome territory. This organization has implications for many biological processes such as chromosomal rearrangements that are common in cancer and the interactions between sub-nuclear structures that control how genes are expressed. Despite this, little is known about the genes or mechanisms that are responsible for creating or maintaining chromosome territories. Here, we show that the Condensin II complex can induce the formation of chromosome territories in fruit flies. We propose that this activity stems from the ability of Condensin II to reduce the length of chromosomes.

## Introduction

It is widely accepted that interphase chromosomes occupy discrete sub-nuclear domains, known as chromosome territories (CTs). CTs have been described in animals, fungi, and plants [1]–[4], suggesting that they may have functional relevance for all eukaryotes. The spatial arrangement of these compartments is non-random and correlates with the functional compartmentalization of the genome, in which actively expressed genes are spatially segregated from silent genes and repetitive DNA sequences [5]–[8]. In light of recent findings, several models of the nucleus posit that CT structure plays a central role in regulating long distance DNA-DNA interactions, heterochromatic gene silencing, DNA repair, and genomic stability [9]–[11]. Despite this, little is known about the factors that regulate the higher levels of chromosome structure or the spatial relationships between different chromosomes.

Several aspects of nuclear architecture can vary across species and cell types, but a few general organizational patterns appear to be highly conserved. Within the nuclei of many mammalian, chicken, and Arabidopsis cells, CTs are roughly spherical and have dimensions that are much smaller than the nuclear diameter [4], [12]–[14]. Alternatively, the chromosomes of budding yeast, cereals, salamanders, and *Ascaris* often exist in polarized, elongated domains known as the Rabl configuration [3], [15]–[17]. This pattern resembles the organization of chromosomes during anaphase: the centromeres of all chromosomes are clustered together at one pole of the nucleus, telomeres are oriented toward the opposite pole, and chromosome arms are similar in length to the diameter of the nucleus [17], [18]. Although chromosomes in the Rabl configuration do occupy discrete regions of the nucleus (i.e. territories), we reserve the term CT to refer specifically to globular structures, such as those of typical mammalian nuclei.

In *Drosophila*, multiple chromosome organization schemes have been well characterized. The Rabl configuration is observed in many diploid nuclei such as those of the early embryo and the larval central nervous system [19], [20]. Polyploid-polytene chromosomes, such as those of the larval salivary gland, also exhibit features of the Rabl configuration [21], whereas spermatocytes have been shown to form non-Rabl, globular CTs in the G2-phase of meiosis I [22]. The polyploid nurse cells of the *Drosophila* ovary are exceptional in several aspects of their nuclear organization and dynamics. Their chromosomes appear polytene during the first few rounds of DNA replication, but they undergo a developmentally regulated reorganization. After the fifth endocycle, homology-dependent pairing interactions are disrupted [23]. In this arrangement known as the “5-blob stage”, each of the 5 major chromosome arms occupies a globular territory, similar in appearance to mammalian CTs. Since nurse cells also have some of the largest nuclei ever described, their structural features are readily observable by light microscopy, making them an attractive model for the study of nuclear architecture.

The molecular mechanisms that control chromosome organization are poorly understood; however, it is clear that Condensin complexes play a central role. In *Drosophila*, there are believed to be two distinct Condensin Complexes. The SMC2/SMC4 heterodimer is common to both Condensin I and Condensin II. Condensin I also contains Cap-G, Cap-D2, and the Kleisin subunit Cap-H, whereas Condensin II contains Cap-D3 and Cap-H2 [24]. While the canonical vertebrate Condensin II also has a Cap-G2 subunit, no Cap-G2 ortholog has been identified in *Drosophila*.

Condensins are ATPases that can alter DNA topology in vitro and have been shown to regulate chromosome structure during mitosis and meiosis [24]–[26]. Recently, it has been shown that Condensins also contribute to the structure of interphase chromosomes [27]. Since Condensin II is required to disrupt nurse cell polytene chromosome pairing near the transition to the 5-blob stage [28], we hypothesized that this complex may also contribute to the forces that drive the formation of globular CTs.

## Results

### Cap-H2 is required for nurse cell chromosome territory formation

**Figure 1:**
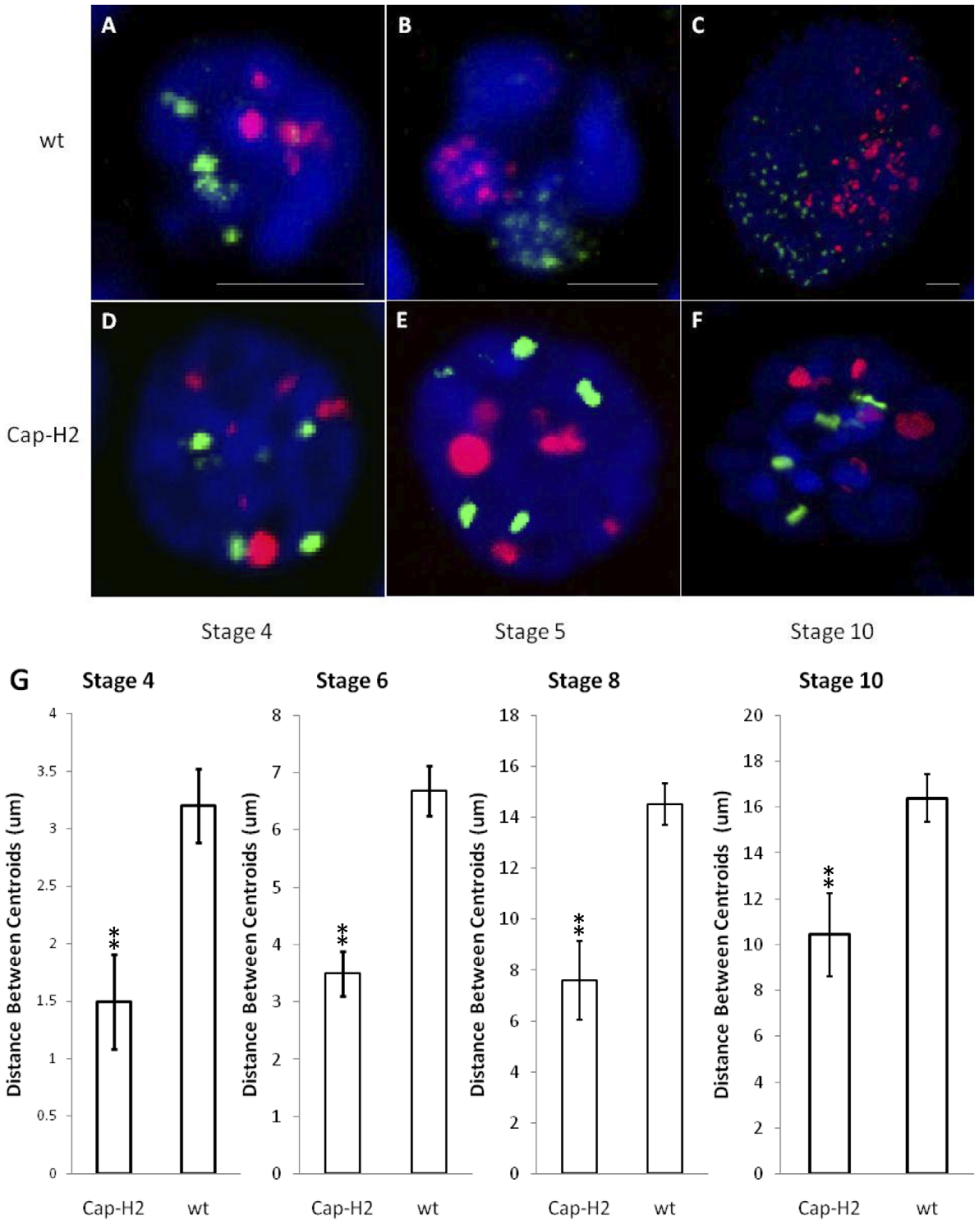
The formation of compact chromosome territories is Condensin II dependent. 3D FISH was performed on nurse cell nuclei to mark the X chromosome in green and the 2^nd^ chromosome in red. DAPI (DNA) is in blue. Representative nuclei from the following genotypes and developmental stages are depicted: A–C) wild type (*y[1]* *w[67c23]*) stages 4, 5, and 10; D–F) *Cap-H2^Z3-0019^/Df(3R)6159* stages 4, 5, and 10. All scale bars are equal to 5 µm. G) Mean distances between X chromosome and 2^nd^ chromosome centroids at stages 4, 6, 8, and 10. Error bars correspond to standard error. One asterisk signifies p<.05 and two asterisks signifies p<.01. Within each category, n = 15.

To assess the impact of Condensin II on the spatial relationships between heterologous chromosomes, we performed FISH (fluorescence in situ hybridization) on wild type and *Cap-H2^Z3-0019^/Df(3R)Exel6159* mutant ovaries using probes to simultaneously mark 12 unique loci: 5 probes spanning the X chromosome were labeled in green and 7 probes on the 2^nd^ chromosome were labeled in red. On average, these probes were spaced ∼5 Mb apart. Confocal microscopy was then used to capture 3D images of nurse cell nuclei. Consistent with previous findings [23], wild type stage 4 nurse cell nuclei appeared polytene and tended to display a single fluorescent focus for each probe (Figure 1A). At stage 5, the chromatids that composed the polytene chromosomes began to unpair from one another and each set of homologous chromosomes became confined to a compact spherical CT (Figure 1B). Initially, each territory was distinguishable by DAPI staining alone due to the presence of a DNA-devoid region called the interchromatin compartment (Figure 1B). At later stages, territories were not apparent by DAPI staining but FISH revealed that CT structure was largely maintained throughout the subsequent endocycles and expansion of the nuclear volume (Figure 1C). In nurse cells carrying mutations in *Cap-H2 (Cap-H2^Z3-0019^/Df(3R)Exel6159)*, a Condensin II subunit, a polytene organization was evident throughout development and compact spherical CTs were not visually apparent at any stage (Figure 1D–1F).

The dispersed FISH signal in wild type nurse cells facilitated the visualization of CT boundaries. In *Cap-H2^Z3-0019^/Df(3R)Exel6159* nurse cell nuclei, this signal was concentrated to a smaller number of foci (Figure 1). Thus, it remained possible that CTs were also present in this genotype but not readily visible. To address this, we computationally segmented the FISH signal from our 3D reconstructions and determined the geometric center of all FISH foci (centroid) for each chromosome-specific set of probes. The distances observed between the centroids of the X and 2^nd^ chromosome probes provided a quantitative measure of the mean spatial separation between these chromosomes. At all stages of development, we found that the X and 2^nd^ chromosomes were significantly more distant from each other in wild type nurse cells than in *Cap-H2^Z3-0019^/Df(3R)Exel6159* (Figure 1G, p<0.01 for each stage). Based on this, we concluded that the compact CT structure observed in wild type nurse cells was dependent upon the presence of Condensin II.

### Condensin II is required to inhibit the Rabl configuration and to disperse heterologous centromeres

We next sought to determine if nurse cell CTs were consistent with the Rabl configuration in which the centromeric regions of all chromosomes cluster together near the nuclear periphery and the telomeres cluster near the opposite pole. We performed FISH using 8 different probes (3 at a time) that targeted loci along the length of the X chromosome. For each of these loci, we determined its mean radial distance from the center of the nucleus. The center of each nucleus was determined based on the geometric center of all DAPI signal. The nuclear radius was then estimated based on the total volume of DAPI signal assuming that each nucleus was a sphere. These estimates correlated well with manual measurements of nurse cell nuclei (R^2^ = .87) and we were unable to detect any differences in total nuclear size in any Condensin II mutant backgrounds (Figure S1). All radial distances are reported as the distance from the center of nucleus divided by the nuclear radius.

**Figure 2:**
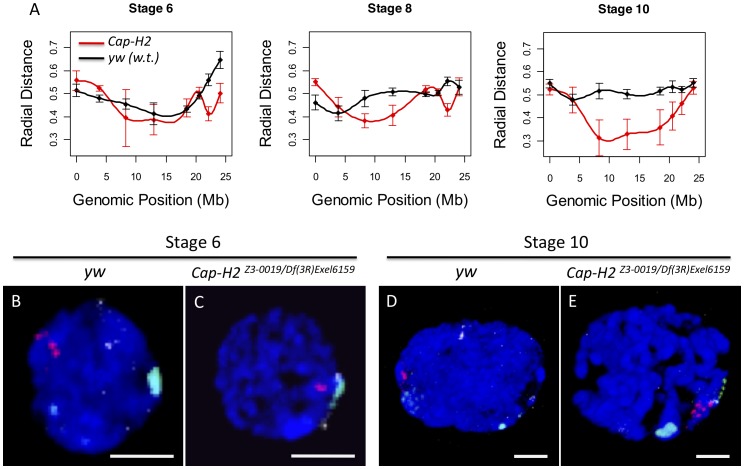
Condensin II inhibits the Rabl conformation. A) For each X chromosome probe, the radial distance from the center of nurse cell nuclei was measured at stages 6, 8, and 10. For each nucleus, the nuclear radius was estimated based on the volume of DAPI staining and the assumption that each nucleus was a sphere. All radial distances are reported as a fraction of the estimated nuclear radius with 0 corresponding to the center of the nucleus and 1 corresponding to the nuclear periphery. Genotypes are indicated by the legend in the bottom panel. Error bars correspond to standard error. B–E) Images of representative nuclei from wild type and *Cap-H2^Z3-0019^/Df(3R)6159* mutant nurse cells. Probes label the pericentric heterochromatin of the X (green), 2^nd^ (red), and 3^rd^ (white) chromosomes.

In wild type nurse cell nuclei from stage 6, 8, and 10 egg chambers, the loci closest to the centromere were consistently near the maximal distance from the center of the nucleus (Figure 2A, Figure S2). Similarly, we observed that the X-chromosome heterochromatin FISH signal in *Cap-H2^Z3-0019^/Df(3R)Exel6159*, *Cap-D3^EY00456^/Df(2L)Exel7023* and *SMC4^k08819^/+; Cap-H2^Z3-0019^/+* nurse cells consistently localized near the nuclear periphery while loci near the middle of the chromosome arm were more centrally located and also exhibited a larger amount of variability in their radial positions (Figure 2A). We next performed 3D-FISH with sets of probes that differentially marked the peri-centric heterochromatin of the X, 2^nd^, 3^rd^, and 4^th^ chromosomes. Again, we observed that peri-centric heterochromatin sequences consistently localized to the nuclear periphery in wild type and *Cap-H2* mutant nuclei (Figure 2B–2E).

**Figure 3:**
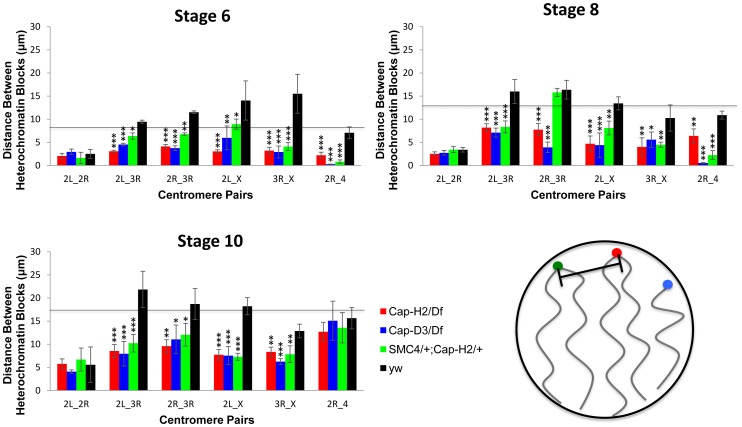
Condensin II promotes the dispersal of heterologous centromeres. The mean distances between the peri-centric microsatellite sequences of the X, 2R, 2L, 3R and 4 chromosome arms at various stages of nurse cell development are shown. The labels below each set of four bars indicate which pair of loci are being compared. The genotypes assayed were *Cap-H2^Z3-0019^/Df(3R)Exel6159, y[1] w[67c23]; P(w[+mC] = lacW)glu[k08819]/+; Cap-H2^Z3-0019^/+*, *Cap-D3^EY00456^/Df(2R)Exel7023*, and wild type (*y[1] w[67c23]*). Error bars correspond to standard errors. One asterisk signifies p<.05, two asterisks signifies p<.01, and three asterisks signifies p<.001. The cartoon in the lower right panel illustrates the distance measurements being made between different heterochromatin FISH signals.

Interestingly, we also observed that while wild type cells had heterochromatic sequences that were dispersed to several loci, the pericentric heterochromatin from all chromosomes was always clustered toward one side of the nucleus in *Cap-H2^Z3-0019^/Df(3R)Exel6159* nurse cells (Figure 2B–2E). Measurement of the distances between these probe centroids confirmed that heterochromatic sequences from different chromosomes remained in close proximity in a *Cap-H2^Z3-0019^/Df(3R)Exel6159* background while these sequences were well separated in wild type nurse cells (Figure 2B–2E, Figure 3). This trend was clear throughout development and was also observed in other allelic combinations of Condensin II subunits indicating that *Cap-H2* was functioning in the context of the Condensin II complex (Figure 3).

Based on these data, we concluded that wild type nurse cell chromosomes exhibit dispersed peri-centric heterochromatin and centromeric regions at several distinct loci near the periphery of the nucleus and this organizational pattern is dependent upon Condensin II. By contrast, in Condensin II mutant nurse cells, all of the heterochromatic sequences and centromeres are clustered together at a single pole, consistent with the Rabl conformation. We lacked the resolution to determine whether the Rabl conformation is present prior to stage 4 so it was not possible to determine if Condensin II disrupts the Rabl configuration or simply prevents it from developing. However, if the Rabl configuration is a vestige of anaphase, then it would be present after the final cell division and require disruption.

### Condensin II is required for axial compaction of nurse cell chromosomes

**Figure 4:**
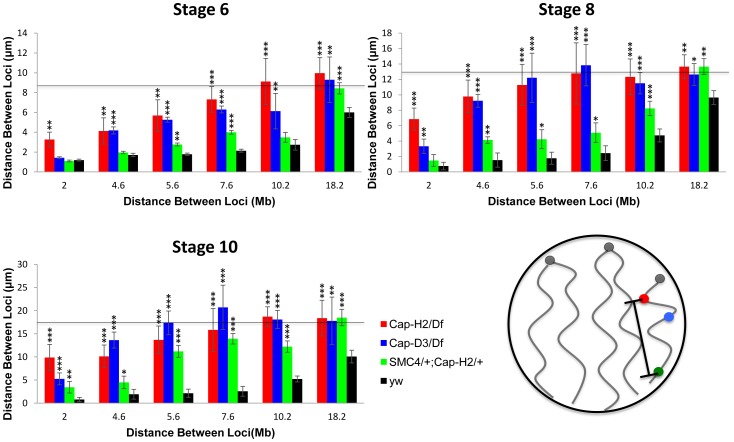
Condensin II promotes chromosome axial compaction. For developmental stage 6, 8, and 10, distances were measured between pairs of loci on the X chromosome. The X-axis indicates the distance between the loci in megabases and the Y-axis indicates their mean spatial separation in microns. The horizontal line indicates the average nuclear radius which is also a good approximation for the mean distance between two random points within a sphere. The following genotypes were assayed: *Cap-H2^Z3-0019^/Df(3R)Exel6159, Cap-D3^EY00456^/Df(2R)Exel7023, y[1] w[67c23]; P(w[+mC] = lacW)glu[k08819]/+; Cap-H2^Z3-0019^/+, wild type (y[1] w[67c23])*. Bars represent standard errors. One asterisk signifies p<.05, two asterisks signifies p<.01, and three asterisks signifies p<.001. The cartoon in the lower right panel illustrates the axial length measurements being made between different loci.

Since Condensins have been implicated in the axial compaction of chromosomes during mitosis and meiosis [29]–[31], we hypothesized that a similar compaction activity was contributing to the formation of nurse cell CTs. To test this, we measured the distances between pairs of locus centroids on the X chromosome in nurse cells with varying degrees of Condensin II activity. We observed that the mean distance between any two X chromosome loci were substantially increased in *Cap-H2^Z3-0019^/Df(3R)Exel6159* nurse cells compared to their wild type counterparts. This trend was consistent and statistically significant for every pair of loci, and every developmental stage, that we analyzed (Figure 4, p<.01). Measurement of the distances between two pairs of probes on the 2^nd^ and 3^rd^ chromosomes showed that these chromosomes were also more compact in the presence of wild-type Condensin II relative to condenisn II mutants (Figure S3, p<0.01). This trend was also evident in double heterozygous *SMC4^08819^/+; Cap-H2^Z3-0019^/+* flies and was consistent when compared to two additional wild type strains, Oregon-R and Canton-S (Figure S3).

It is interesting to note that the different levels of compaction in different Condensin II allelic combinations correlates well with their respective severity in polytene unpairing phenotypes (Figure 4, Figures S4 and S5). These data indicated that Condensin II functions to promote axial compaction in nurse cell chromosomes and that this compaction activity is concomitant with its polytene unpairing activity.

We next sought to estimate the magnitude of chromosome axial compaction induced by Condensin II. Since the distance between two loci is strictly limited by the diameter of the nucleus, which is much smaller than the length of a nurse cell polytene chromosome, the most accurate estimates of actual chromosome length come from pairs of probes that delimit short chromosomal segments. At stage 4, two loci on the X-chromosome spanning 2 Mb were separated by an average distance of 1.18±0.12 µm in wild type nurse cells. In later developmental stages, the mean distance between these probes remained fairly constant despite a dramatic increase in the size of the nucleus. By stage 10, the distance between these probes actually appeared to have been reduced to 0.80±0.44 µm, though this difference was not statistically significant (Figure 4). In *Cap-H2^Z3-0019^/Df(3R)Exel6159* nurse cells, chromosome length consistently increased throughout development. At stage 4 the same 2 loci were found to be 2.17±0.22 µm apart, on average. By stage 10, this distance had increased to 9.88±2.82 µm (Figure 4) indicating that Condensin II was responsible for at least a 10-fold reduction of chromosome length. In a few of the *Cap-H2^Z3-0019^/Df(3R)Exel6159* nuclei, it was possible to trace the path of the polytene chromosome between the two probes. Direct measurement of the curved path determined its length to be 12.87±0.46 µm (n = 3) (Figure S6). Based on this, we estimate that Condensin II can induce a 12- to 16-fold reduction in nurse cell chromosome length.

Extrapolation from this estimate of compaction would mean that the entire X chromosome may be as short as 10 µm in wild type stage 10 nurse cells. This is only twice the length of the mitotic X chromosome, which is approximately 5 µm long in diploid cells (data not shown). This suggested that Condensin II was inducing compaction of interphase nurse cell chromosomes, just as it has been proposed to do during mitosis. However, it remained a formal possibility that Condensin II was indirectly contributing to chromosomal compaction or functioning to maintain chromosome length rather than reduce it, per se.

### Cap-H2 overexpression in salivary glands is sufficient to induce axial compaction and CT formation

**Figure 5:**
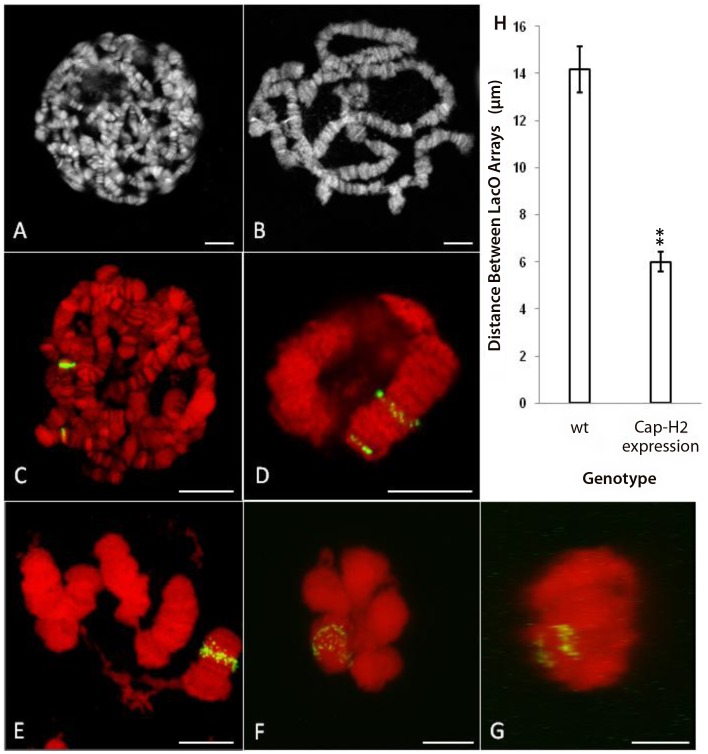
Expression of *Cap-H2* in salivary glands induces axial shortening of chromosomes. A) DAPI stained, unspread salivary gland chromosome squash from wild type larva raised at 25°C. B) DAPI stained, unspread salivary gland squash from hsp70>*Gal4*/UAS>*Cap-H2* raised at 25°C. C) Projection of salivary gland nucleus from Hs83>LacI-GFP/+; 50F-LacO/60F-LacO with 1 hr, 37°C heat shock. DAPI is shown in red and GFP signal is shown in green. D) Projection of salivary gland nucleus from Hs83>LacI-GFP/+; 50F-LacO/60F-LacO; hsp70>*Gal4*/UAS>*Cap-H2* with 1 hr, 37°C heat shock. E) A unique example (same treatment as in D) in which the nuclear envelope has ruptured revealing details of chromosome structure. F) Projection of salivary gland nucleus from Hs83>LacI-GFP/+; 50F-LacO/60F-LacO; hsp70>*GAL4*/UAS>*Cap-H2* with 1 hr, 37°C heat shock. Both LacO arrays remain confined within a common territory during the dispersal process. G) A projection of the same nucleus in F through the Y-axis showing the separation between the two LacO arrays. H) Measurements of the distance between LacO insertions in cases similar to C and D. All scale bars correspond to 10 µm.

To further assess the ability of Condensin II to compact interphase chromosomes, we ectopically expressed *Cap-H2* in the larval salivary glands. Larvae carrying multiple, heat shock inducible, *Cap-H2* transgenes exhibit completely unpaired salivary gland chromosomes after a 24 hour heat shock [28]. Since this phenotype resembled the dispersal observed in wild type nurse cell chromosomes, we suspected that it was also associated with chromosome axial compaction, just as in nurse cells. In flies carrying a single *Cap-H2* transgene, we found that polytene unpairing was limited while chromosome length was substantially reduced compared to wild type flies, even without heat shock (Figure 5A–5B). This is presumably due to weak expression of *Cap-H2* at 25°C. To quantify this phenotype, we crossed in chromosomes that contained insertions of LacO arrays at cytological bands 50F (near the center of chromosome arm 2R) and 60F (near the telomere of 2R) as well as a LacI-GFP fusion protein that was also heat shock inducible. The result was a fly line that displayed two fluorescent chromosomal loci and expressed *Cap-H2* in response to heat shock.

In a control line that contained the LacI-GFP transgene and the two LacO arrays without a *Cap-H2* transgene, a 1-hour heat shock resulted in polytene salivary gland chromosomes with two fluorescent bands that were separated by a mean distance of 14.2±1.0 µm (Figure 5C, 5H). With the addition of the *Cap-H2* transgene and identical treatment, most GFP bands started to become unpaired but the two LacO arrays typically remained spatially distinct from each other (Figure 5D). In cases such as these, the mean distance between the LacO arrays was reduced to 6.0±0.4 µm (Figure 5H). Extrapolation from this figure suggested that the entire right arm of chromosome 2 was close to 12 µm long after expression of *Cap-H2*. In some instances, entire salivary gland chromosome arms could be resolved and measured, supporting this estimate (Figure 5E). A previous study determined that the entire right arm of chromosome 2 was 144.3±7.8 µm long in wild type salivary glands [21]. Based on this, we concluded that Condensin II is capable of inducing at least 12-fold compaction of salivary gland chromosomes.

Taken together, our observations in nurse cells and salivary glands indicated that Condensin II can induce a tremendous amount of axial compaction in interphase chromosomes. Moreover, this compaction coincides with the formation of compact CTs in both cell types (Figure 1B, Figure 5F–5G). Prior to the dispersal of salivary gland polytene chromosomes, each chromosome arm became compacted into a discrete nuclear compartment, similar to the 5-blob stage in nurse cells (Figure 1B, Figure 5F–5G). Even after substantial dispersal had occurred, GFP signal from both LacO insertion sites remained confined within a fraction of the nuclear volume (Figure 5F–5G). The finding that Condensin II can produce such remarkably similar phenotypes in two unrelated cell types suggests that this complex is likely to regulate chromosome length and CT structure in other contexts as well. This idea is further supported by the observation that meiotic CT formation in non-polyploid *Drosophila* primary spermatocytes is Condensin II dependent [26].

## Discussion

We have shown that in the polyploid nurse cells of *Drosophila*, Condensin II activity is required to unpair polytene chromosomes and disperse heterochromatic sequences. Interestingly, while dispersal of heterologous peri-centric heterochromatin blocks on different chromosomes was dependent on Condensin II activity, homologous (or allelic) heterochromatic sequences tended to be paired and localized to the nuclear periphery in both wild-type and Condensin II mutant flies (Figure 2, Figure 3, Figures S2 and S4). Although Condensin II promotes the unpairing of euchromatic homologous sequences, unpaired homologs and sister chromatids were sequestered into discrete territories within the nucleus (Figure 1, Figure S5D). We also observed that axial compaction of chromosomes in nurse cells required Condensin II activity (Figure 4, Figure S3) and overexpression of *Cap-H2* in salivary gland cells could induce shortening of chromosome arms (Figure 5). These observations are consistent with previous work showing that Condensin II serves to individualize homologous chromosomes by disrupting pairing interactions in polytene, diploid, and meiotic cells [26], [28]. In addition, a recent whole genome RNAi screen in cultured *Drosophila* cells also identified *Cap-H2*, *Cap-D3* and *SMC2* as homolog pairing disruptors, further supporting an anti-pairing activity for Condensin II [32].

If the activity of Condensin II spatially separates homologous chromosomes from each other, how does it simultaneously keep all homologous chromosomes of a polyploid nucleus confined within a common territory? One likely reason is that unpairing of homologs and sister chromatids is incomplete in polyploid cells. When we measured the unpairing of several loci in nurse cells, we found that pairing was disrupted at all euchromatic loci we examined, but heterochromatic regions near the telomere and centromere retained some associations with their allelic sequences (Figures S4, S7). We speculate that this incomplete dispersal of nurse cell polytene chromosomes is due, in part, to under-replicated heterochromatic regions associated with telomeres and centromeres [23], [33]–[34]. In addition to under-replicated regions, it is also possible that nurse cell pairing of homologous peri-centric heterochromatin is actively maintained and insensitive to Condensin II activities, while clustering of heterologous peri-centric heterochromatin is inhibited by Condensin II activities.

**Figure 6:**
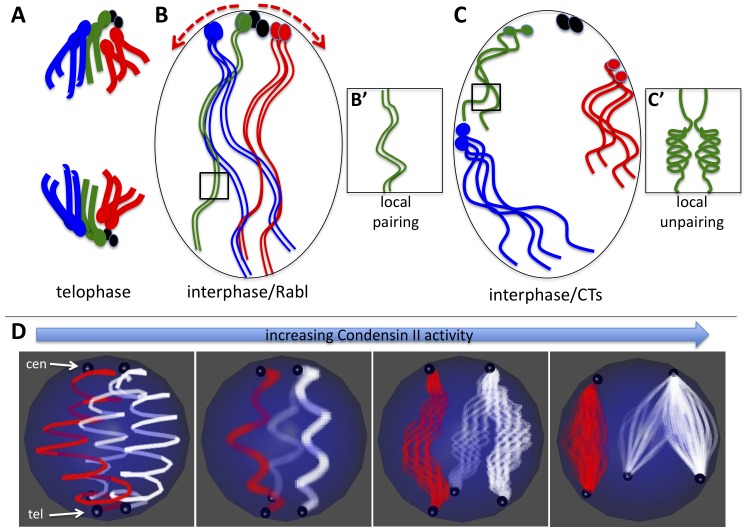
Model for Condensin II–mediated chromosome territory formation. A) Condensed mitotic chromosomes are pulled to opposite poles and centromeres cluster at the poles in telophase. Four chromosomes are shown where the ovals represent centromeres and/or peri-centric heterochromatin. The small fourth chromosomes are shown in black. B) In interphase, decondensed chromosomes adopt the Rabl configuration with clustered centromeres simply because of their previous organization in telophase. B') Long and relaxed interphase homologous chromosomes are paired. C) Activation of Condensin II in interphase nuclei has two direct consequences: First it induces axial compaction of the chromosome arms. Second, in order to accommodate the shorting distance between telomeres and centromeres, heterochromatin slides along the nuclear periphery as the axial length of the arms decrease (see red arrows in B). Because heterochromatin proteins interact with the nuclear periphery, we speculate that as euchromatic regions of chromosome arms shorten they are drawn to the nuclear periphery. Heterochromatin and other chromatin interactions with the nuclear periphery combined with the Condensin mediated shortening of chromosome arms forces chromosomes into discrete territories. C') Chromosome arm compaction is most likely accompanied by chromatin folding and coiling that occludes homologous sequences from interacting in *trans*. D) The model in A–C depicts a diploid cells scenario, but the same mechanical principles can apply to a polyploid nucleus that forms CTs. In the panel on the left, two polytene chromosomes (the X chromosome in red and an autosome in white) are shown where centromeres (cen) are clustered at the North pole and telomeres (tel) are clustered at the South pole of the nucleus. As Condensin II activity increases (left to right) the chromatin fibers within polytenes begin to unpair as the chromosome arms shorten. Note that although euchromatic sequences unpair, homologous heterochromatin blocks remain paired and move in concert. By contrast, heterologous heterochromatin blocks disperse to different parts of the nuclear periphery. As above (B–C), Condensin II mediated compaction drives globular CT formation at the nuclear periphery as a consequence of chromatin interactions with nuclear envelope proteins.

Previous studies have found that centromeric and telomeric sequences tend to localize to the nuclear periphery in a number of *Drosophila* cell types as well as in other organisms [20]–[21], [35]. We found that the same is true in nurse cells (Figure 2). Interestingly, in wild type nurse cells, all loci on the X chromosome tended to move toward the nuclear periphery with developmental progression (Figure 2A). In the polytene nurse cell chromosomes of *Cap-H2^Z3-0019^/Df(3R)Exel6159* mutants, heterochromatic sequences remained near the periphery but euchromatic loci were always located more centrally (Figure 2A). If heterochromatic loci are physically tethered to the nuclear envelope, then one would expect chromosomal compaction to pull linked, euchromatic loci toward the nuclear periphery as well. Centromeres and telomeres on the same chromosome would likely move toward each other by sliding along the nuclear envelope (Figure 6).

We propose a general model for the effects of Condensin II activity on interphase chromosome structure and the spatial organization of chromosomes within the nucleus: Interphase chromosomes may first adopt a Rabl configuration upon exit from mitotic telophase, simply because all centromeres are pulled to the spindle pole body and thus are clustered (Figure 6A–6B). In interphase cells, Condensin II promotes a dramatic axial compaction of chromosomes (Figure 4, Figure 5) and this compaction is likely associated with topological changes in DNA. This Condensin II mediated compaction of chromatin (Figure 6C) sequesters DNA into discrete chromosomal structures, as proposed for mitotic chromosome condensation [36].

Sequestering of sequences into intrachromosomal folds disfavors interchromosomal interactions between homologous loci (Figure 6C'), which disrupts polytene chromosome structure and trans-sensitive processes such as transvection [28]. Pairing between allelic, heterochromatic loci in polyploid nurse cells is unaffected by Condensin II and these sequences localize to the nuclear periphery in nurse cells (Figure 2, Figure S7). The combination of Condensin II mediated chromosome compaction and heterochromatic interactions with the nuclear periphery drive each chromosome into a globular CT that is associated with the nuclear envelope (Figure 6D). Because homologous heterochromatic sequences in polyploid nurse cells are under-replicated we speculate that this keeps the unpaired chromatids confined together within the CT. Thus, we propose that Condensin II serves to spatially separate individual chromosomes by inhibiting homology dependent pairing interactions as well as interactions between heterologous chromosomes. Many aspects of this model may be applicable to the roles of Condensins in the nuclear reorganization that occurs during mitosis or meiosis, since it seems to contribute similarly to the processes of chromosome compaction and individualization in all of these contexts [26], [28].

The relationship between chromosome individualization and compaction is currently unclear. Since Condensin II always appears to promote both processes in nurse cells and salivary gland cells, it may be that they both stem from a common enzymatic activity. It is tempting to propose that compaction drives individualization since the former is always detectable first. However, FISH does not provide sufficient resolution to distinguish between DNA sequences that are physically interacting and those that are no longer interacting at the molecular level but remain in close proximity simply because they were paired in the recent past. Perhaps DNA-DNA interactions in *cis* and in *trans* compete with each other, as proposed by Wu and colleagues [37]–[38]. In this paradigm, Condensin II could function to shift the equilibrium away from *trans* and toward *cis* interactions. This model would not only explain the temporal coincidence of compaction and polytene dispersal, but also the curious observation that polytene chromosome length increases with ploidy (Figure 4) [21]. In the latter case, homologous chromosomes interact with each other in *trans* (i.e. pair) and thus occlude intrachromosomal interactions that would otherwise restrict chromosome length. More copies of homologous sequences would shift the equilibrium in favor of more pairing interactions, thus further increasing chromosome length.

With regards to the generality of the proposed model, it is important to consider the cell types that it is based on. While nurse cells are germline derived and salivary glands are somatic, both tissues are polyploid. Though polyploidy is the rule rather than the exception in terminally differentiated *Drosophila* tissues, it may be that certain aspects of this model do not apply to diploid cells. We have not yet been able to compare chromosome axial lengths in wild-type and Condensin II mutant diploid cells, but efforts are underway to achieve this. The strongest evidence for the generality of our model comes from observations in spermatogenesis. During prophase I, wild-type primary spermatocytes form very well separated CTs, each of which is associated with the nuclear envelope. However, this organization is completely absent in Condensin II mutants which appear to contain a chromocenter-like structure of dense DAPI signal [26]. The phenotypes in this diploid cell type are strikingly similar to our observations in nurse cells suggesting that similar mechanisms may regulate genomic architecture regardless of ploidy.

Though the cytological effects of Condensin II on chromosome structure are clear, the molecular mechanisms that produce these changes remain enigmatic. *In vitro* evidence suggests that it mostly likely involves changes in DNA topology such as DNA supercoiling or looping [25]. Given that Condensin I and Condensin II share a common set of SMC subunits, it can be difficult to tease apart their effects. Our results agree well with recent reports that Condensin II is an important regulator of mitotic chromosome axial compaction while condensin I controls lateral compaction [39]–[40]. It seems likely that the phenotypes displayed after overexpression of Cap-H2 in salivary glands are the result of shifting an equilibrium toward Condensin II at the expense of Condensin I since Cap-H2 is specific to Condensin II.

Regardless, we believe this is the first report of any gene that directly controls interphase CT structure in any system. It will be of great interest to determine if Condensins function to regulate the formation or maintenance of CTs in other eukaryotes. A recent report described structural defects in the interphase nuclei of mouse embryonic stem cells when Condensin subunits were knocked down by RNAi [27]. Perhaps these cells exhibit disorganized CT structure or increased chromosome length.

Though it is clear that the spatial positions of genes, within a nucleus, can influence their expression patterns [41]–[42], it is not well understood how CTs contribute to gene regulation. We have now described two reciprocal systems in which we can prevent or induce Condensin II mediated CT formation and observe the effects. Future studies will be directed at elucidating the causal relationships between nuclear organization and transcriptional activity.

Considering the effects that Condensin II has on large scale chromosome conformations and nuclear organization, perhaps it would promote long distance interactions between sequences on the same chromosome while precluding interactions between elements on different chromosomes. This is supported by the finding that transvection is enhanced in Condensin II mutants and suppressed when *Cap-H2* is overexpressed [28]. It is known that the transcriptional profiles of many genes correlate with those of their chromosomal neighbors [43]. We speculate that Condensin II may contribute to the mechanisms of co-regulation in gene neighborhoods as it is likely to promote the spatial co-localization of genetically linked genes and/or long-distance enhancer-promoter *cis* interactions.

## Materials and Methods

### Fly strains

The following fly strains were used in this study: *y[1] w[1118]* (wild type control for all nurse cell studies, unless otherwise noted), *y[1] w[67c23]; P(w[+mC] y[+mDint2] = EPgy2)Cap-D3[EY00456]/Df (2L)Exel7023 (Cap-D3* mutant), *y[1] w[67c23]; P(w[+mC] = lacW)glu[k08819]/+;* Cap*-H2^Z3-0019^/+ (SMC4, Cap-H2* double heterozygote), *Cap-H2^Z3-0019^/Df(3R)Exel6159* (*Cap-H2* mutant), *Oregon-R-S* (wild type control for all salivary gland studies), w[*]; P(w[+mC] = GAL4-nos.NGT)40 P(w[+mC] = lacO.256x)43 P(lacO.256x)50F P(lacO.256x)57A P(lacO.256x)60AB/CyO; P(w[+mC] = UAS-GFP.lacI)1.2/TM3, Sb[1] (lacO array 50F insertion line), w[*]; P(w[+mC] = lacO.256x)60F (lacO array 60F insertion line). *Canton-S* and *Oregon-R* (OrR) wild type controls were also used in Figure S2. All flies were maintained at 25°C on standard cornmeal molasses media.

### Labeling of probes

BAC clones were selected to span the X chromosome at regular intervals. The following BACs were ordered from CHORI BACPAC Resources: BACR25p24, BACR18c23, BACR23m08, BACR32h11, BACR33k15, BACR32l12. These map approximately to 3.9, 8.3, 12.9, 18.5, 20.5, and 22.1 Mb, respectively. To mark the heterochromatic region of the X chromosome we synthesized PCR products to the 359 bp repeat sequence using the primers 5′-CGGTCATCAAATAATCATTTATTTTGC-3′ and 5′-CGAAATTTGGAAAAACAGACTCTGC-3′. We also used the following oligonucleotides to label the 2L, 2R, 3R, and 4 heterochromatic regions, respectively: (AATAG)_5_, (AACAC)_5_, 5′-CCCGTACTGGTCCCGTACTGGTCCCGTACTCGGTCCCGTACTCGGT-3′, and (AATAT)_5_. Telomeric probes were made by PCR amplification of plasmids containing a 2 kb *ApaI–ApaI* fragment of the 3′ UTR of HeT-A and the *23Zn-1* fragment containing ORF1+ORF2 of HeT-A.

Midipreps were performed on bacterial cultures to isolate BAC DNA. In some cases, probes were made by whole genome amplification (Sigma WGA kit) of previously isolated BAC DNA. 20 µg of BAC DNA or amplified DNA was digested overnight with the restriction enzymes *AluI*, *HaeIII*, *MseI*, *MspI*, and *RsaI*. DNA fragments were purified using a Qiagen PCR cleanup column, ethanol precipitated, and end labeled with aminoallyl dUTP (Invitrogen ARES Alexa Fluor DNA Labeling Kit) using terminal deoxytransferase as previously described [28]. DNA fragments were purified again on a PCR cleanup column (Qiagen) and ethanol precipitated. These labeled DNA fragments were then conjugated to Alexa488, Alexa546, or Alexa647 dyes as described in the ARES DNA labeling kits. Labeled probes were purified on a PCR cleanup column, ethanol precipitated, and resuspended in 20 µL hybridization buffer.

### Fluorescence in situ hybridization and imaging

FISH protocol was adapted primarily from Dernburg et al., 2000. 0–2 day old female flies were fattened on yeast with males at 25°C for 2 days. 5 ovary pairs were dissected in Grace's medium and fixed within 15 minutes. Fixation was performed for 4 minutes in 100 mM Sodium cacodylate, 100 mM sucrose, 40 mM Sodium acetate, 10 mM EGTA with 3.7% formaldehyde. Ovaries were washed twice for 5 minutes each in 2X SSCT and individual ovarioles were separated in 2X SSCT. Ovarioles were washed for 10 minutes each in 2X SSCT with 20%, 40%, and 50% formamide at room temperature and once more for 2 hours in 2X SSCT with 50% formamide at 30°C (for AT rich heterochromatic probes) or 37°C. 200–500 ng of probe was mixed into a total of 40 µL hybridization buffer, denatured at 95°C, and snap frozen in liquid nitrogen. Formamide mixture was aspirated from ovarioles and replaced with probe/hybridization buffer mixture. This suspension was mixed gently and incubated at 30 or 37C for 10 minutes prior to a 2 min denaturation at 92°C. Tubes were immediately returned to hybridization oven and left overnight at the specified temperature.

The next day, ovarioles were washed 4 times for 30 minutes each in 2X SSCT with 50% formamide at the hybridization temperature. Ovarioles were then washed 10 minutes each in 2X SSCT with 40% and 20% formamide. Three 5 minute washes with 2X SSCT were performed to remove the remaining formamide. The ovarioles were then stained with 10 ng/mL DAPI in 2X SSCT for 10 minutes and washed twice with 2X SSCT for 10 minutes each. Ovarioles were mounted in vectashield. Pieces of number 1½ coverslips were used as spacers between the actual coverslip and slide to prevent flattening of the egg chambers. Slides were imaged on a Zeiss 510 meta confocal microscope using a 1.4 NA, 63× objective.

### Nurse cell image analysis

Individual channels were separated from 3D image stacks and analyzed using the 3D Object Counter plugin for imageJ. The spatial coordinate and intensity measurements were then analyzed in the R programming environment. Intensity thresholds were set as follows: 25 for DAPI, 35 for Alexa488 and Alexa647, 55 for Alexa546. Objects smaller than 10 voxels were ignored. Spot counts for each probe are simply the number of objects detected. For each probe in each nucleus, the centroid was defined as the center of total fluorescence intensity. Principal component analysis was performed using point weights equal to the total fluorescence intensity of each object. The center of fluorescence intensity in the DAPI channel was set as the nuclear center and radial distances for each object were measured in relation to this point.

### Salivary gland imaging and measurements

Transgenic lines carrying a 256-repeat array of the Lac-O sequence at chromosomal position 50F were crossed to lines carrying the same Lac-O array inserted at position 60F with transgenes Hsp70>*Gal4* and UAS>*Cap-H2*, as previously described [28]. These lines also had the ability to express a GFP-LacI fusion protein that binds to the LacO arrays and marks the chromosomal insertion site of the LacO array. Expression of GFP-LacI and *Cap-H2* was controlled with heatshock at 37°C, as detailed in the text and figure legends. GFP-LacI in salivary glands was imaged as previously described [28]. 3D distances between the two Lac-O arrays were measured as described for nurse cell images.

## Supporting Information

Figure S1 Nuclear Size is Not Affected in Condensin Mutants. Box-and-whisker plots of nurse cell nuclear radii. A–C) For each genotype, at each developmental stage, individual nuclear radii were calculated based of the volume of DAPI signal in confocal stacks assuming the nuclei are spherical. These calculations consistently underestimate the true radius since some regions within the nucleus show DAPI staining at background level. D–F) Nuclear radii were also estimated based on direct measurement. A single z-slice was selected from a confocal stack for which the x–y area of a given nucleus was near the maximum. A circle was then superimposed over the slice of the nucleus and manually adjusted to best fit the outer boundary of DAPI signal. The boxes show the first quartile, median, and third quartile. The whiskers correspond to 95% confidence intervals using the method of Chambers et al. 1983.
(TIF)

Figure S2 Radial positions of X chromosome loci. For each X chromosome probe, the mean radial distance from the center of nurse cell nuclei was measured at stages 6, 8, and 10. For each nucleus, the nuclear radius was estimated based on the volume of DAPI staining and the assumption that each nucleus was a sphere. All radial distances are reported as a fraction of the estimated nuclear radius with 0 corresponding to the center of the nucleus and 1 corresponding to the nuclear periphery. Genotypes are indicated by the legend in the bottom panel and correspond to *Cap-H2^Z3-0019^/Df(3R)Exel6159*, *Cap-D3* ^EY00456^/Df(2R)Exel7023, y[1] w[67c23]; P(w[+mC] = lacW)glu[k08819]/+; *Cap-H2^Z3-0019^/+*, and wild type (y[1] w[67c23]). Error bars represent standard errors.
(TIFF)

Figure S3 Condensin II induces compaction of the 2^nd^ and 3^rd^ chromosomes in nurse cells. Two loci on chromosome 2 (the histone locus at 21.4 Mb) and band 34D at 13.8 Mb on 2L) were probed in *y[1]* *w[67c23]; P(w[+mC] = lacW)glu[k08819]/*+; *Cap-H2^Z3-0019^/+*, and wild type (OrR) ovaries. The distances between these loci were measured in stage 8 nurse cell nuclei and the mean distances are plotted. Two loci on the chromosome 3 (*Cap-H2* at 6.6 Mb and Ubx at12.5 Mb on 3R) were probed in *Cap-H2^Z3-0019^/Df(3R)Exel6159* and wild type (*Canton-S*) ovaries. The distances between these loci were measured in stage 10 nurse cell nuclei and the mean distances are plotted. Bars represent standard errors. Two asterisks indicates p<.01.
(TIFF)

Figure S4 Condensin II is necessary for unpairing of nurse cell polytene chromosomes. Mean number of distinct fluorescent foci (spots) in nurse cell nuclei throughout development. Data from stage 6, 8, and 10 nurse cell nuclei are shown. Dispersal of the X chromosome telomere (0 Mb) was inferred by dividing the total number of telomeric spots by 5 (the mean number of spots seen in salivary gland polytene chromosome squashes). Error bars correspond to standard errors.
(TIFF)

Figure S5 The effects of Condensin II on nurse cell chromosome organization. 3D FISH was performed to label three loci on the X chromosome (12.9 Mb-Red, 18.5 Mb-White, 20.5 Mb-Green). Representative nuclei from stage 10 egg chambers are displayed. (A) *Cap-H2^Z3-0019^/Df(3R)Exel6159*, (B) *Cap-D3^EY00456^/Df(2R)Exel7023*, (C) *y[1]* *w[67c23]; P(w[+mC] = lacW)glu[k08819]/+; Cap-H2^Z3-0019^/+*, (D) wild type (*y[1]* *w[67c23]*). The scale bar represents 10 µm for all images.
(TIFF)

Figure S6 Estimation of polytene chromosome length. A–B) Examples of stage 10 nurse cell nuclei from *Cap-H2^Z3-0019^/Df(3R)Exel6159* mutant ovaries where the path of the polytene chromosome between two probes was clearly visible. The green probe marks the locus at 20.5 Mb and the red probe marks the locus at 18.5 Mb on the X-chromosome. The length of the 2 Mb region spanning these two loci was measured to be approximately 12.9±0.56 µm (n = 3). Scale bars equal 10 µm.
(TIFF)

Figure S7 Unpairing of heterochromatic loci in nurse cells. FISH was performed with probes corresponding to microsatellite sequences on all 4 chromosomes. The mean number of spots for each probe is depicted for nurse cell nuclei at stages 6, 8, and 10. Genotypes are indicated by the legend in the bottom panel and correspond to *Cap-H2^Z3-0019^/Df(3R)Exel6159*, *Cap-D3^EY00456^/Df(2R)Exel7023*, *y[1] w[67c23]; P(w[+mC] = lacW)glu[k08819]/+; Cap-H2^Z3-0019^/+*, and wild type (*y[1] w[67c23]*). Error bars correspond to standard error.
(TIFF)
