# Supplementary figures and images for: Condensin II Promotes the Formation of Chromosome Territories by Inducing Axial Compaction of Polyploid Interphase Chromosomes
Source: PLoS Genet. 2012 Aug 30;8(8):e1002873. doi: 10.1371/journal.pgen.1002873 (PMC3431300; doi:10.1371/journal.pgen.1002873)

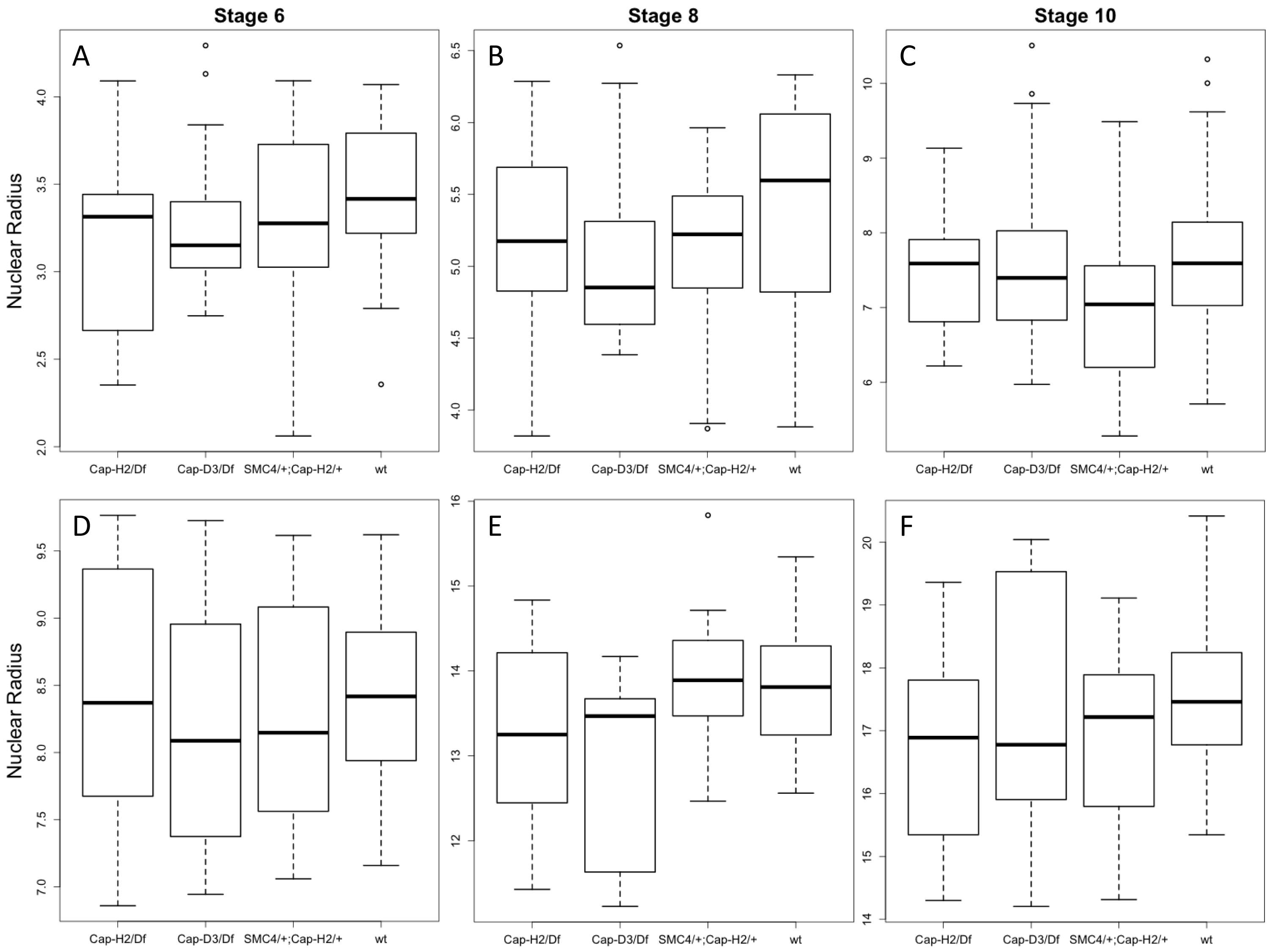

Supplement: Figure S1 — Nuclear Size is Not Affected in Condensin Mutants. Box-and-whisker plots of nurse cell nuclear radii. A–C) For each genotype, at each developmental stage, individual nuclear radii were calculated based of the volume of DAPI signal in confocal stacks assuming the nuclei are spherical. These calculations consistently underestimate the true radius since some regions within the nucleus show DAPI staining at background level. D–F) Nuclear radii were also estimated based on direct measurement. A single z-slice was selected from a confocal stack for which the x–y area of a given nucleus was near the maximum. A circle was then superimposed over the slice of the nucleus and manually adjusted to best fit the outer boundary of DAPI signal. The boxes show the first quartile, median, and third quartile. The whiskers correspond to 95% confidence intervals using the method of Chambers et al. 1983. (TIF) [file pgen.1002873.s001.tif]

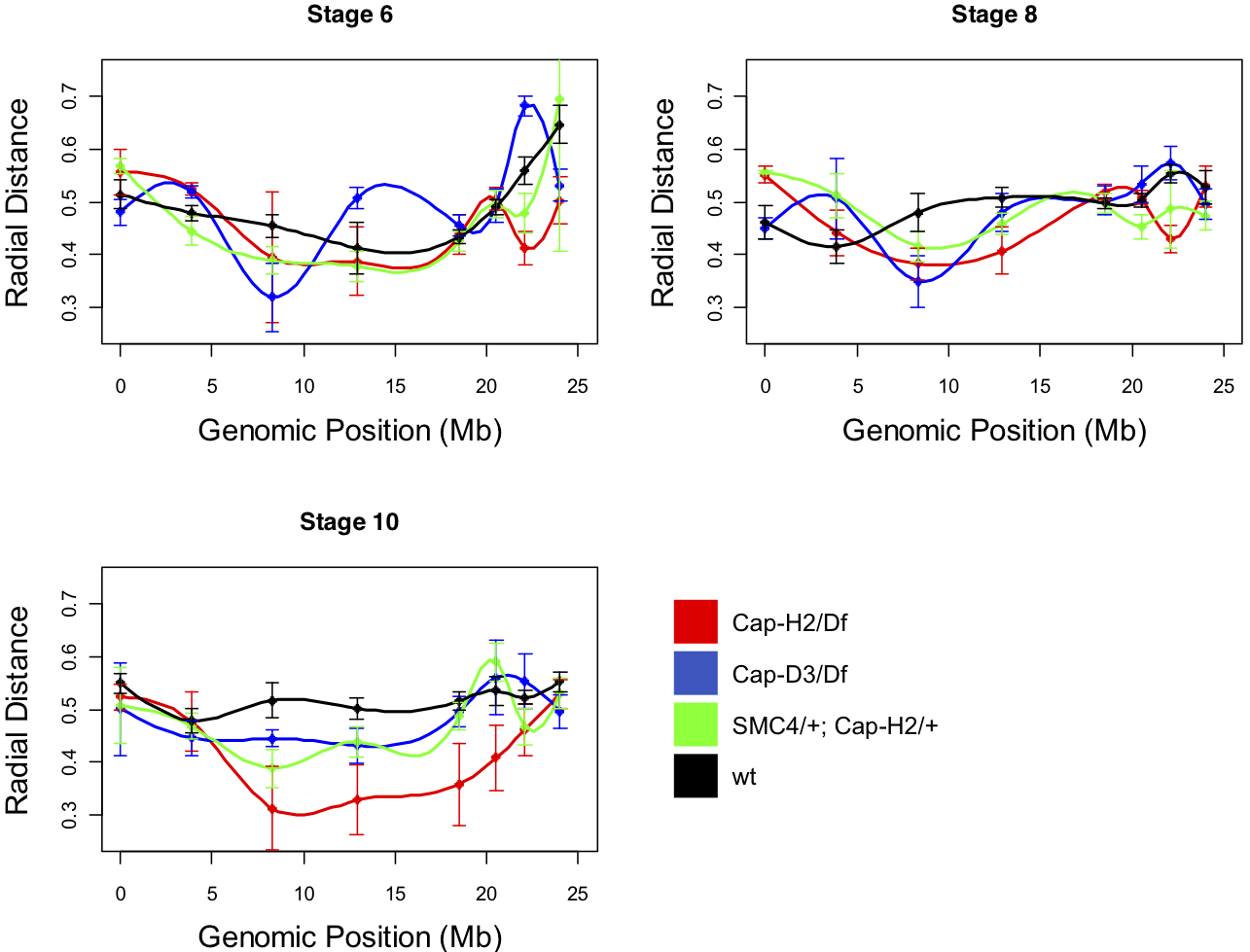

Supplement: Figure S2 — Radial positions of X chromosome loci. For each X chromosome probe, the mean radial distance from the center of nurse cell nuclei was measured at stages 6, 8, and 10. For each nucleus, the nuclear radius was estimated based on the volume of DAPI staining and the assumption that each nucleus was a sphere. All radial distances are reported as a fraction of the estimated nuclear radius with 0 corresponding to the center of the nucleus and 1 corresponding to the nuclear periphery. Genotypes are indicated by the legend in the bottom panel and correspond to Cap-H2Z3-0019/Df(3R)Exel6159, Cap-D3 EY00456/Df(2R)Exel7023, y[1] w[67c23]; P(w[+mC] = lacW)glu[k08819]/+; Cap-H2Z3-0019/+, and wild type (y[1] w[67c23]). Error bars represent standard errors. (TIFF) [file pgen.1002873.s002.tiff]

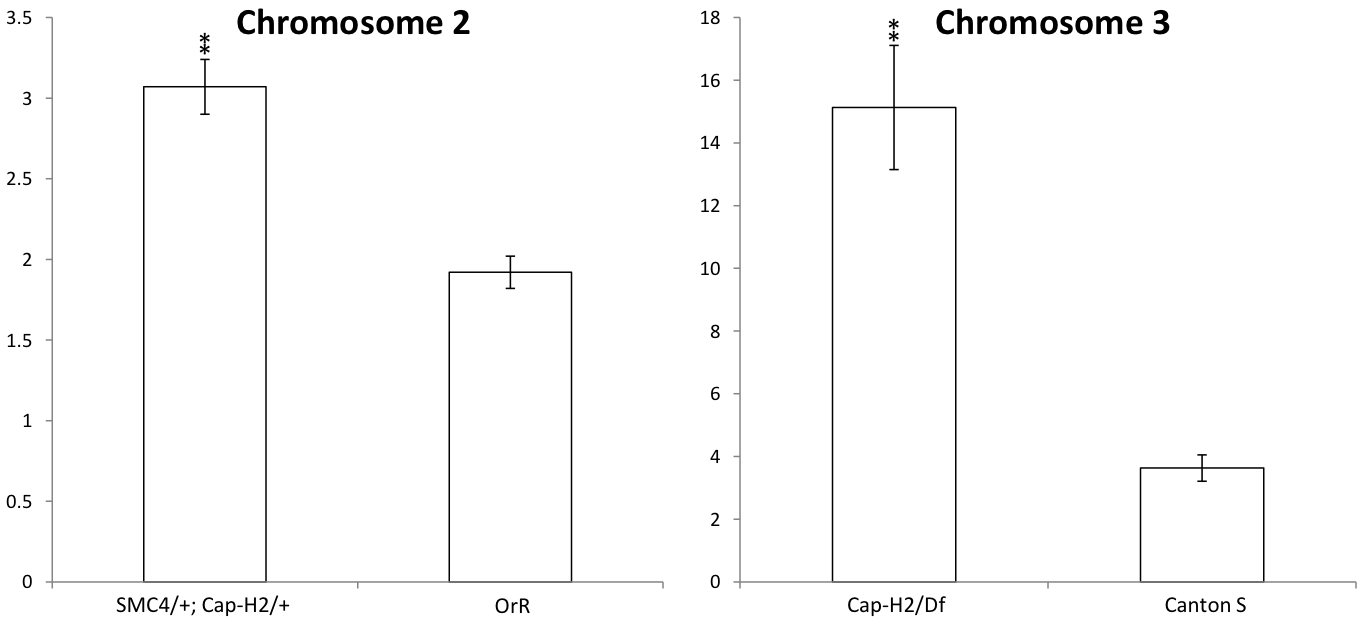

Supplement: Figure S3 — Condensin II induces compaction of the 2nd and 3rd chromosomes in nurse cells. Two loci on chromosome 2 (the histone locus at 21.4 Mb) and band 34D at 13.8 Mb on 2L) were probed in y[1] w[67c23]; P(w[+mC] = lacW)glu[k08819]/+; Cap-H2Z3-0019/+, and wild type (OrR) ovaries. The distances between these loci were measured in stage 8 nurse cell nuclei and the mean distances are plotted. Two loci on the chromosome 3 (Cap-H2 at 6.6 Mb and Ubx at12.5 Mb on 3R) were probed in Cap-H2Z3-0019/Df(3R)Exel6159 and wild type (Canton-S) ovaries. The distances between these loci were measured in stage 10 nurse cell nuclei and the mean distances are plotted. Bars represent standard errors. Two asterisks indicates p<.01. (TIFF) [file pgen.1002873.s003.tiff]

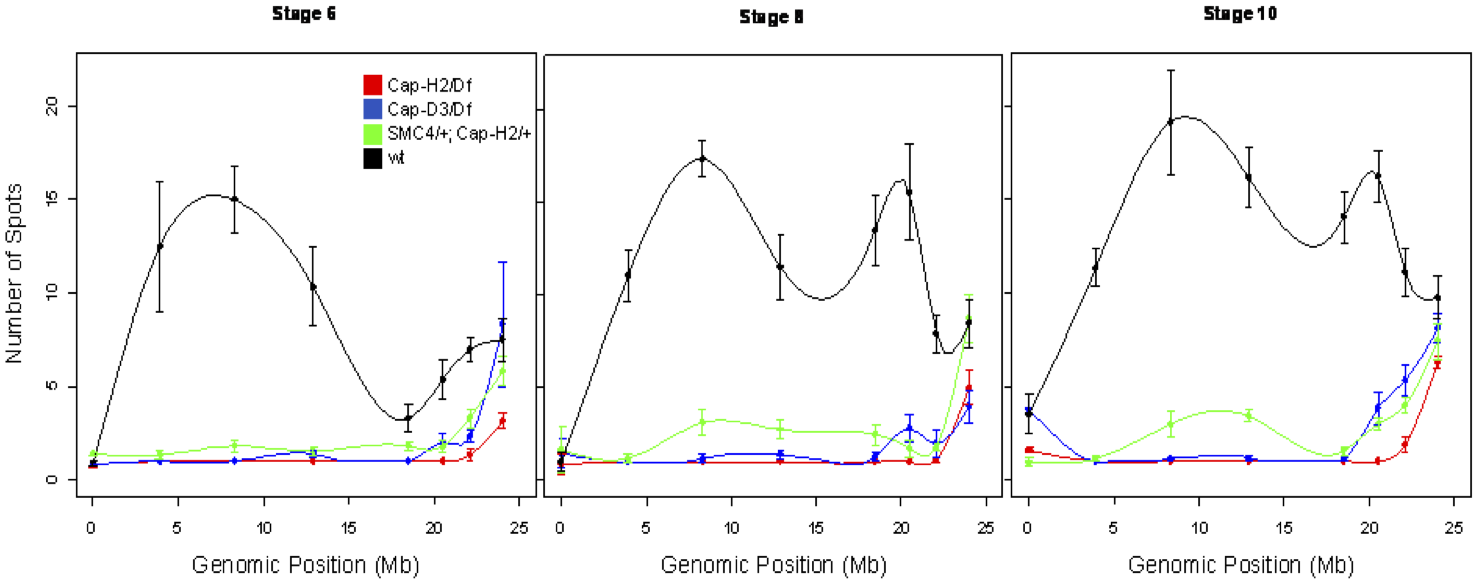

Supplement: Figure S4 — Condensin II is necessary for unpairing of nurse cell polytene chromosomes. Mean number of distinct fluorescent foci (spots) in nurse cell nuclei throughout development. Data from stage 6, 8, and 10 nurse cell nuclei are shown. Dispersal of the X chromosome telomere (0 Mb) was inferred by dividing the total number of telomeric spots by 5 (the mean number of spots seen in salivary gland polytene chromosome squashes). Error bars correspond to standard errors. (TIFF) [file pgen.1002873.s004.tiff]

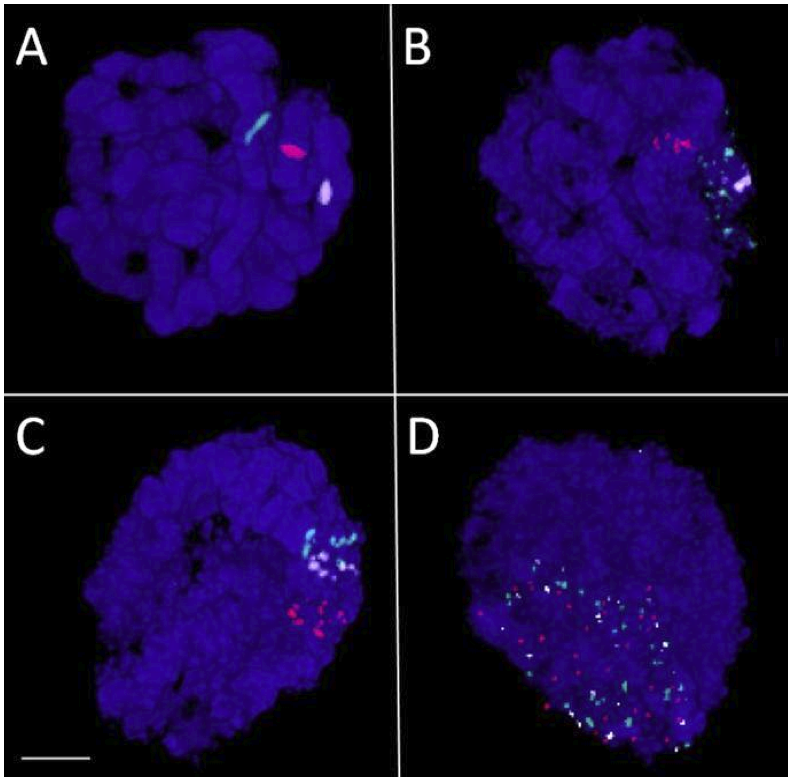

Supplement: Figure S5 — The effects of Condensin II on nurse cell chromosome organization. 3D FISH was performed to label three loci on the X chromosome (12.9 Mb-Red, 18.5 Mb-White, 20.5 Mb-Green). Representative nuclei from stage 10 egg chambers are displayed. (A) Cap-H2Z3-0019/Df(3R)Exel6159, (B) Cap-D3EY00456/Df(2R)Exel7023, (C) y[1] w[67c23]; P(w[+mC] = lacW)glu[k08819]/+; Cap-H2Z3-0019/+, (D) wild type (y[1] w[67c23]). The scale bar represents 10 µm for all images. (TIFF) [file pgen.1002873.s005.tiff]

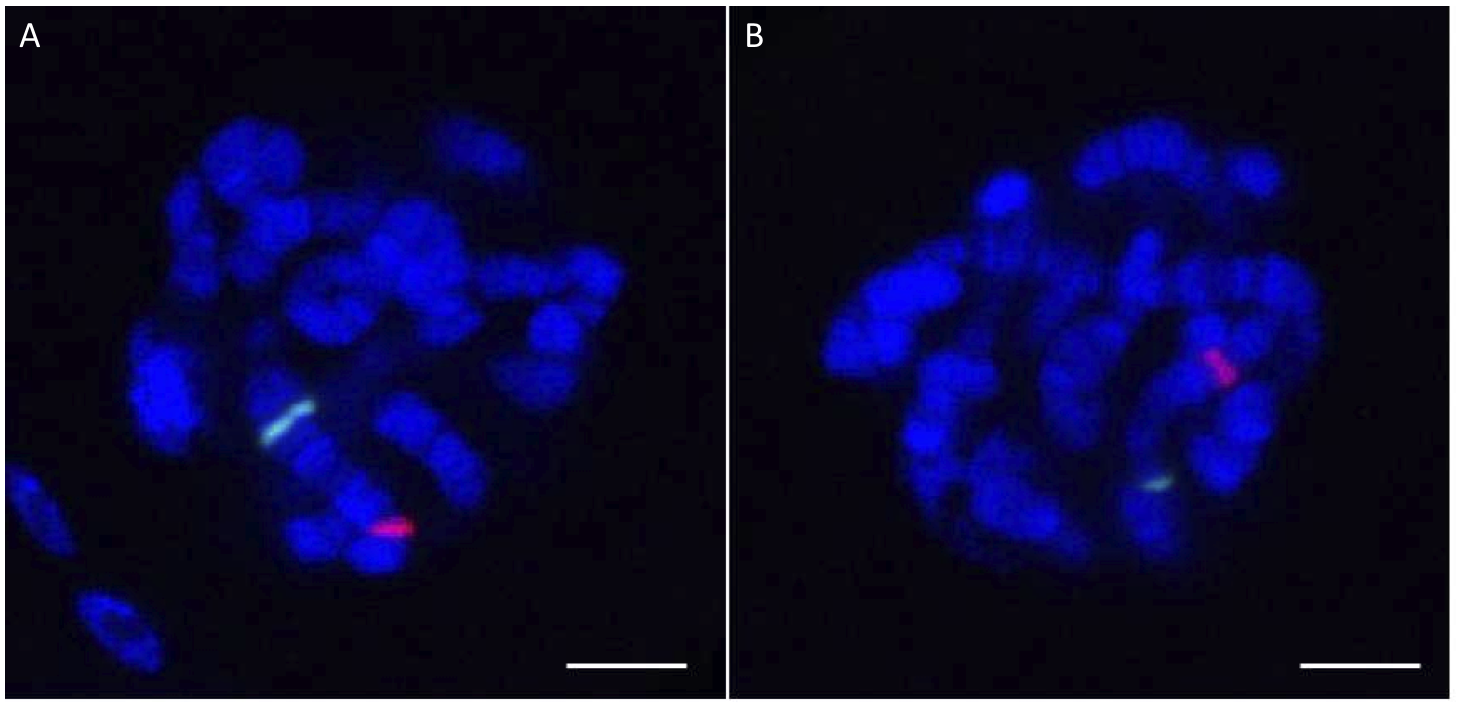

Supplement: Figure S6 — Estimation of polytene chromosome length. A–B) Examples of stage 10 nurse cell nuclei from Cap-H2Z3-0019/Df(3R)Exel6159 mutant ovaries where the path of the polytene chromosome between two probes was clearly visible. The green probe marks the locus at 20.5 Mb and the red probe marks the locus at 18.5 Mb on the X-chromosome. The length of the 2 Mb region spanning these two loci was measured to be approximately 12.9±0.56 µm (n = 3). Scale bars equal 10 µm. (TIFF) [file pgen.1002873.s006.tiff]

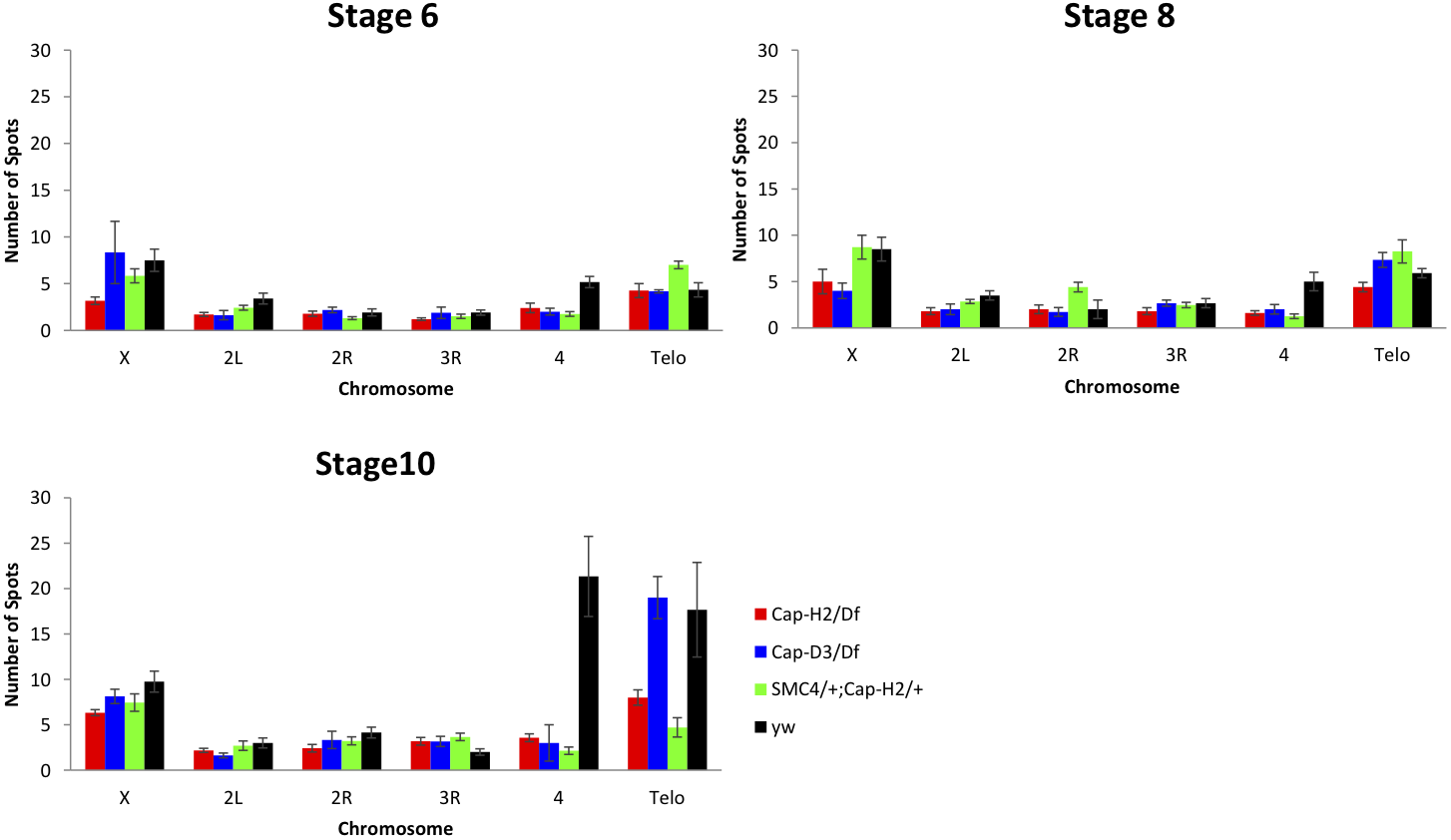

Supplement: Figure S7 — Unpairing of heterochromatic loci in nurse cells. FISH was performed with probes corresponding to microsatellite sequences on all 4 chromosomes. The mean number of spots for each probe is depicted for nurse cell nuclei at stages 6, 8, and 10. Genotypes are indicated by the legend in the bottom panel and correspond to Cap-H2Z3-0019/Df(3R)Exel6159, Cap-D3EY00456/Df(2R)Exel7023, y[1] w[67c23]; P(w[+mC] = lacW)glu[k08819]/+; Cap-H2Z3-0019/+, and wild type (y[1] w[67c23]). Error bars correspond to standard error. (TIFF) [file pgen.1002873.s007.tiff]
